# Utility of virtual monoenergetic images from spectral detector computed tomography in improving image segmentation for purposes of 3D printing and modeling

**DOI:** 10.1186/s41205-019-0038-y

**Published:** 2019-01-18

**Authors:** Elias Kikano, Nils Grosse Hokamp, Leslie Ciancibello, Nikhil Ramaiya, Christos Kosmas, Amit Gupta

**Affiliations:** 1 Department of Radiology, University Hospitals Cleveland Medical Center/Case Western Reserve University, 11100 Euclid Ave, Cleveland, OH 44106 USA; 2 Institute for Diagnostic and Interventional Radiology, University Hospital Cologne, Cologne, Germany

**Keywords:** 3D printing, Spectral detector CT, Segmentation, Dual-energy CT, Dual layer CT

## Abstract

**Background:**

One of the key steps in generating three-dimensional (3D) printed models in medicine is segmentation of radiologic imaging. The software tools used for segmentation may be automated, semi-automated, or manual which rely on differences in material density, attenuation characteristics, and/or advanced software algorithms. Spectral Detector Computed Tomography (SDCT) is a form of dual energy computed tomography that works at the detector level to generate virtual monoenergetic images (VMI) at different energies/ kilo-electron volts (keV). These VMI have varying contrast and attenuation characteristics relative to material density. The purpose of this pilot project is to explore the use of VMI in segmentation for medical 3D printing in four separate clinical scenarios. Cases were retrospectively selected based on varying complexity, value of spectral data, and across multiple clinical disciplines (Vascular, Cardiology, Oncology, and Orthopedic).

**Results:**

In all four clinical cases presented, the segmentation process was qualitatively reported as easier, faster, and increased the operator’s confidence in obtaining accurate anatomy. All cases demonstrated a significant difference in the calculated Hounsfield Units between conventional and VMI data at the level of targeted segmentation anatomy. Two cases would not have been feasible for segmentation and 3D printing using conventional images only. VMI data significantly reduced conventional CT artifacts in one of the cases.

**Conclusion:**

Utilization of VMI from SDCT can improve and assist the segmentation of target anatomy for medical 3D printing by enhancing material contrast and decreasing CT artifact.

**Electronic supplementary material:**

The online version of this article (10.1186/s41205-019-0038-y) contains supplementary material, which is available to authorized users.

## Background

The workflow for medical three-dimensional (3D) printing is consistent and involves initial acquisition of the imaging data, segmentation of anatomy, 3D mesh post-processing, and physical 3D printing [1, 2]. One of the most time consuming and limiting factors throughout this workflow is at segmentation [3, 4]. Computer software tools such as density thresholding, automatic region growing, edge detection, and manual segmentation rely on a combination of the user’s and software’s ability to differentiate various densities as well as advanced pixel-based software algorithms [5].

Dual-energy computed tomography has become clinically available in the mid-2000s [6, 7]. There are several different approaches available that can be categorized into emission- and detection-based systems [8, 9]. While the first group comprises several concepts (e.g. dual source computed tomography, kVp-switching computed tomography), there is only one technology clinically available that works on the detector level and does not require protocol selection prior to the scan: Spectral Detector Computed Tomography (SDCT). It is equipped with a dual-layer detector that registers high and low energy photons in the lower and upper, detector layer, respectively [10].

From a physical perspective, attenuation in CT imaging can be considered as the sum of the photoelectric-effect and Compton-scattering. While the former is predominant in energies up to 100 keV and mostly dependent on the atomic number of any given material, the later depends on the physical density and becomes predominant in energies > 100 keV [11].Besides other image reconstructions, dual energy image registration allows for computation of so-called virtual monoenergetic images (VMI). These are available in a range of range of 40–200 keV using linear blending and extrapolation of information from both detector layers.

VMI approximate images are acquired with a true monoenergetic X-ray [12]. Hence, low keV VMI accentuate differences regarding the material atomic number resulting in an increase in soft-tissue contrast. This is why iodinated contrast media is frequently administered in CT imaging due to its high atomic number (Z = 53) [11]. In addition, k-edge effects further enhance the iodine-associated attenuation. Photons with an energy in the proximity of an elements k-edge are more likely to be absorbed. Since the k-edge of iodine lies at 33 keV, photons with 40 keV are more likely to be absorbed resulting in increased attenuation [11]. High keV VMI can be used to reduce image artifacts due to photon starvation [13–15]. These characteristics are highlighted in Additional file 1: Figure S1.

Based on these physics and the dual-energy CT technology available, the combination of conventional and VMI data may facilitate segmentation for 3D printing. This study aimed to investigate if VMI from SDCT accelerates and/or improves image pre-processing and segmentation for medical 3D printing.

## Methods

Multiple cases were acquired using a clinical SDCT scanner (IQon, Philips Healthcare, Best, The Netherlands) with different imaging protocols. Institutional Review Board (IRB) approval was obtained (NHR-17-57) for retrospective evaluation and four SDCT cases were reviewed for relevant anatomy. Cases were selected based on complexity, value of spectral data compared to associated conventional images, and clinical specialty (Vascular, Cardiology, Oncology, and Orthopedic). Virtual monoenergetic images (VMI) from the original scans at multiple energy levels were obtained. The spectral reconstructions are available for every scan done on the SDCT scanner and all examinations were performed for clinical indications. No scan was acquired for the sole purpose of this study.

Image segmentation was carried out using built-in software tools that come with the vendor’s proprietary image viewer (IntelliSpace Portal (ISP), v9.0, Best, The Netherlands). Both conventional and VMI at 40 to 170 keV at 10 to 20 keV increments were processed and reviewed for segmentation (40 keV, 50 keV, 70 keV, 90 keV, 100 keV, 120 keV, 130 keV, 150 keV, and 170 keV). For cases 1, 2, and 3, the 40 keV images were ultimately utilized due to the closeness to the k-edge value of iodine (33.2 keV) which provided for maximum contrast effect. For case 4, 120 keV data was utilized for metal artifact reduction.

The institution’s single 3D lab senior person completed the requested anatomical segmentation for each of the cases (Additional file 1: Figure S2). Subjective qualitative feedback regarding the performance of segmentation tools was collected by the single person performing the segmentation and differential in calculated Hounsfield Units for relevant anatomy was compared between conventional and VMI.

Additional generation of the Standard Tessellation Language (STL) models was completed in ISP. Postprocessing of the STL files was completed in Autodesk Meshmixer prior to printing. Considerations were made for model orientation and support materials required for 3D printing including removing free-floating elements, proper hollow construction, and maximizing build space. All 3D printed models were made using the Formlabs Form 2 stereolithography (SLA) printer with standard resin material.

## Results

### Case 1: Transcatheter aortic valve replacement pre-procedure planning for vascular access simulation

**Fig. 1:**
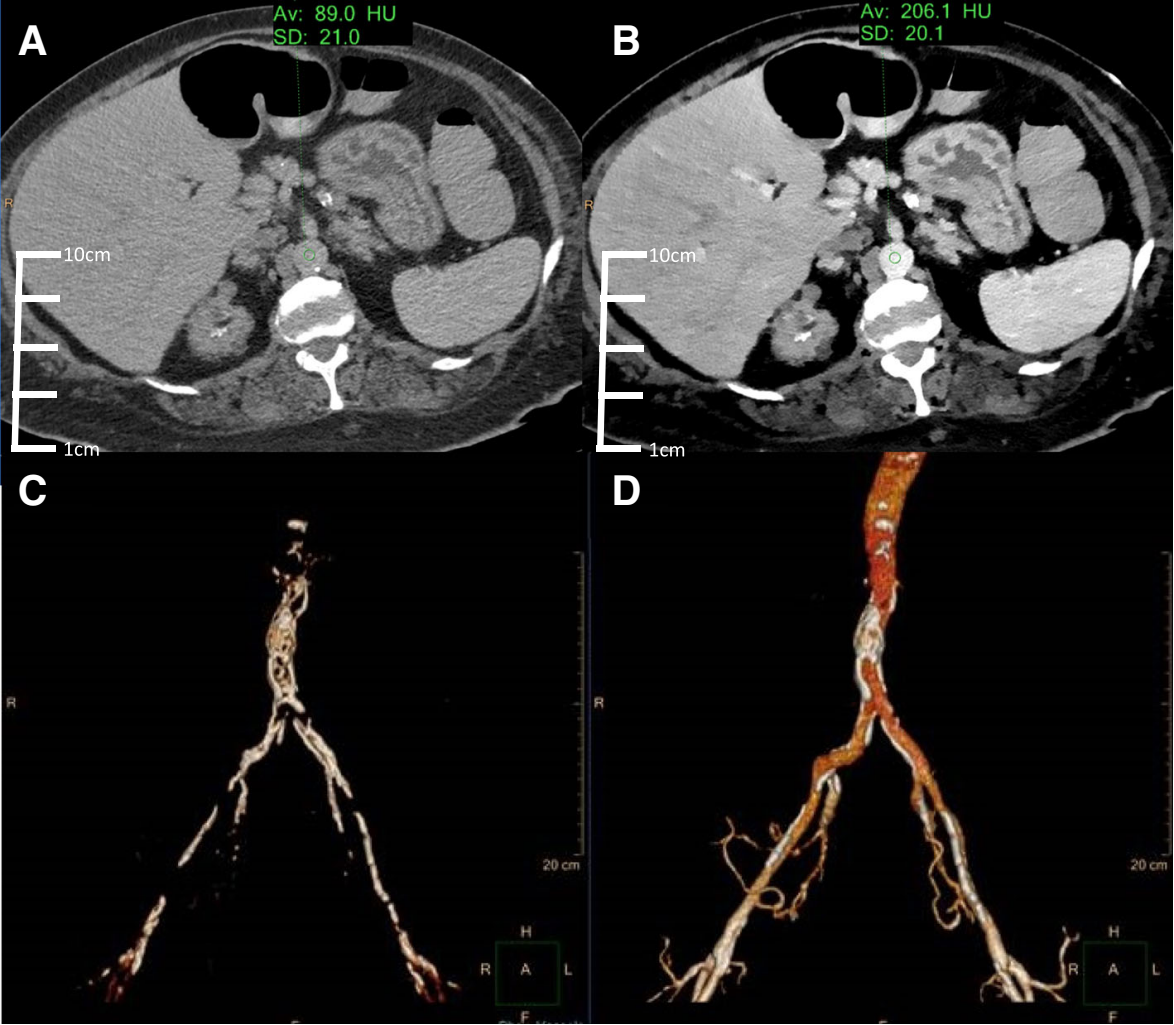
Conventional (**a**) and VMI 40 keV (**b**) axial SDCT images of case 1 TAVR planning. The 40 keV VMI data demonstrates increased aortic vascular contrast enhancement (HU: 206) compared to conventional CT (HU: 89). 3D volume renderings of the abdominal aorta from the conventional (**c**) and 40 keV VMI (**d**) data created using the same segmentation tools and workflow show better continuity and inclusion of the vascular lumen on 40 keV VMI compared to conventional CT

71-year-old female with a history of renal failure on dialysis who presented for transcatheter aortic valve replacement (TAVR) evaluation. Part of the routine preprocedural TAVR workup includes assessing the abdominal aortic vasculature for extent of atherosclerotic disease. The patient’s body mass index was 28. As per the department low contrast dose protocol, the patient was injected with 25 mL Isovue 370 at a rate of 4 mL/sec followed by a 40 mL saline chase. Bolus tracking technology was used to trigger the scan once enhancement reaches 10 Hounsfield Units (HU) over baseline. Images in Fig. 1 are reconstructed in both conventional and virtual monoenergetic images at 40 keV.

**Fig. 2:**
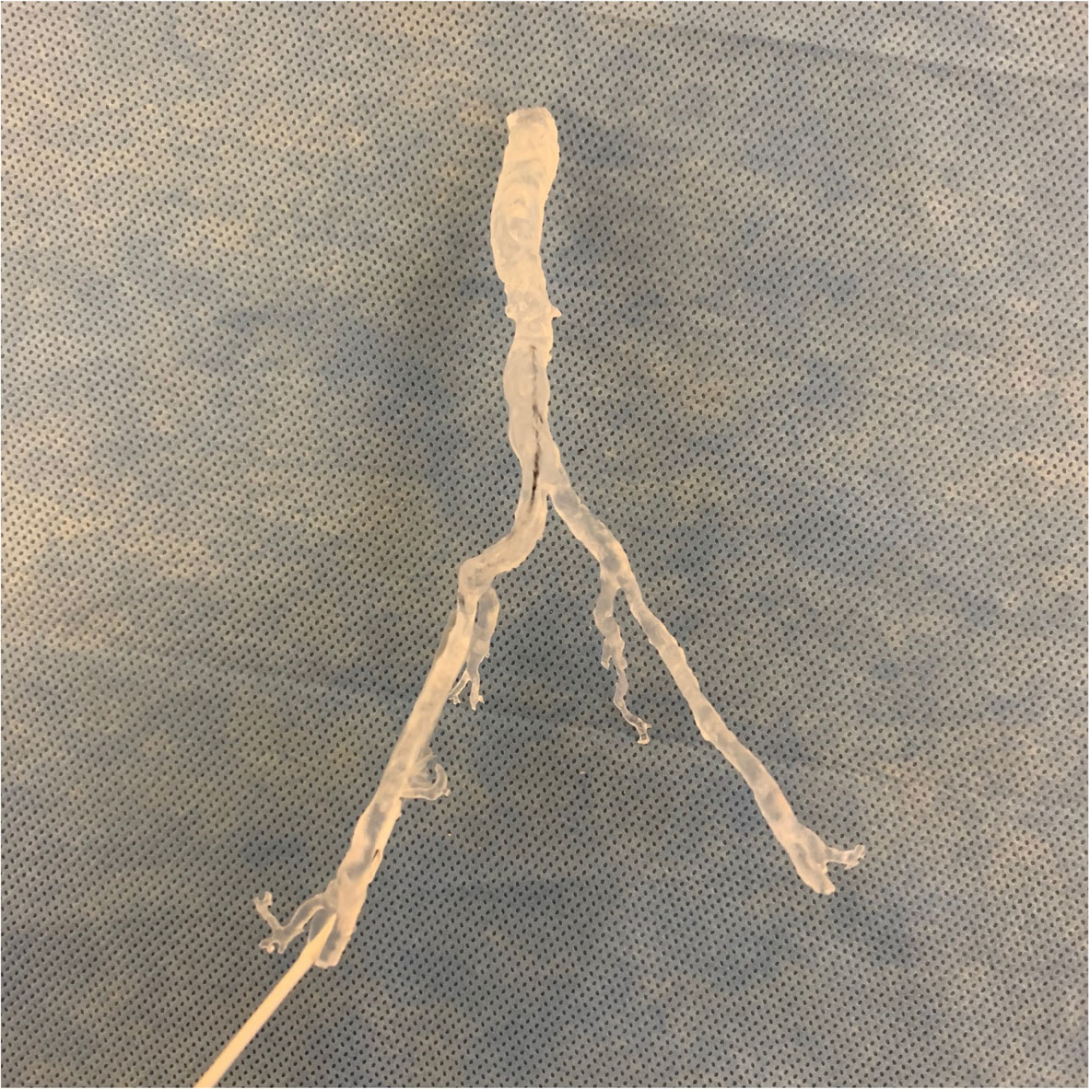
3D printed aortic vasculature from the 40 keV VMI data at 25% scale size. The model was printed using the Formlabs Form 2 SLA 3D printer with standard clear resin material. A guide wire is placed through the right common femoral artery simulating vascular access

The abdominal aorta vascular anatomy was targeted for segmentation. Despite the low volume contrast bolus, the low 40 keV VMI data demonstrated a greater than two-fold increase in HU of the abdominal vessels compared to conventional CT. A combination of thresholding and iodine mapping segmentation tools were utilized. These tools generated a more accurate 3D volume rendered model of the abdominal vasculature lumen using the 40 keV VMI data compared to conventional CT with minimal manual segmentation required. The final 3D printed model of the abdominal vasculature lumen using the VMI at 40 keV was designed and completed for vascular access simulation (Fig. 2).

### Case 2: Left atrial appendage Thrombus segmentation for purposes of patient education

77-year-old male with a history of atrial fibrillation who presented for preprocedural planning for left atrial appendage closure device. Part of the preprocedural workup includes helical cardiac CT with retrospective gating. The scan delay is a threshold/trigger-based hybrid with the scanner manually started once the user sees the first blush of contrast in the right atrium. The patient received 25 mL of iodinated contrast Isovue 370 at 4 mL/sec followed by a saline bolus.

**Fig. 3:**
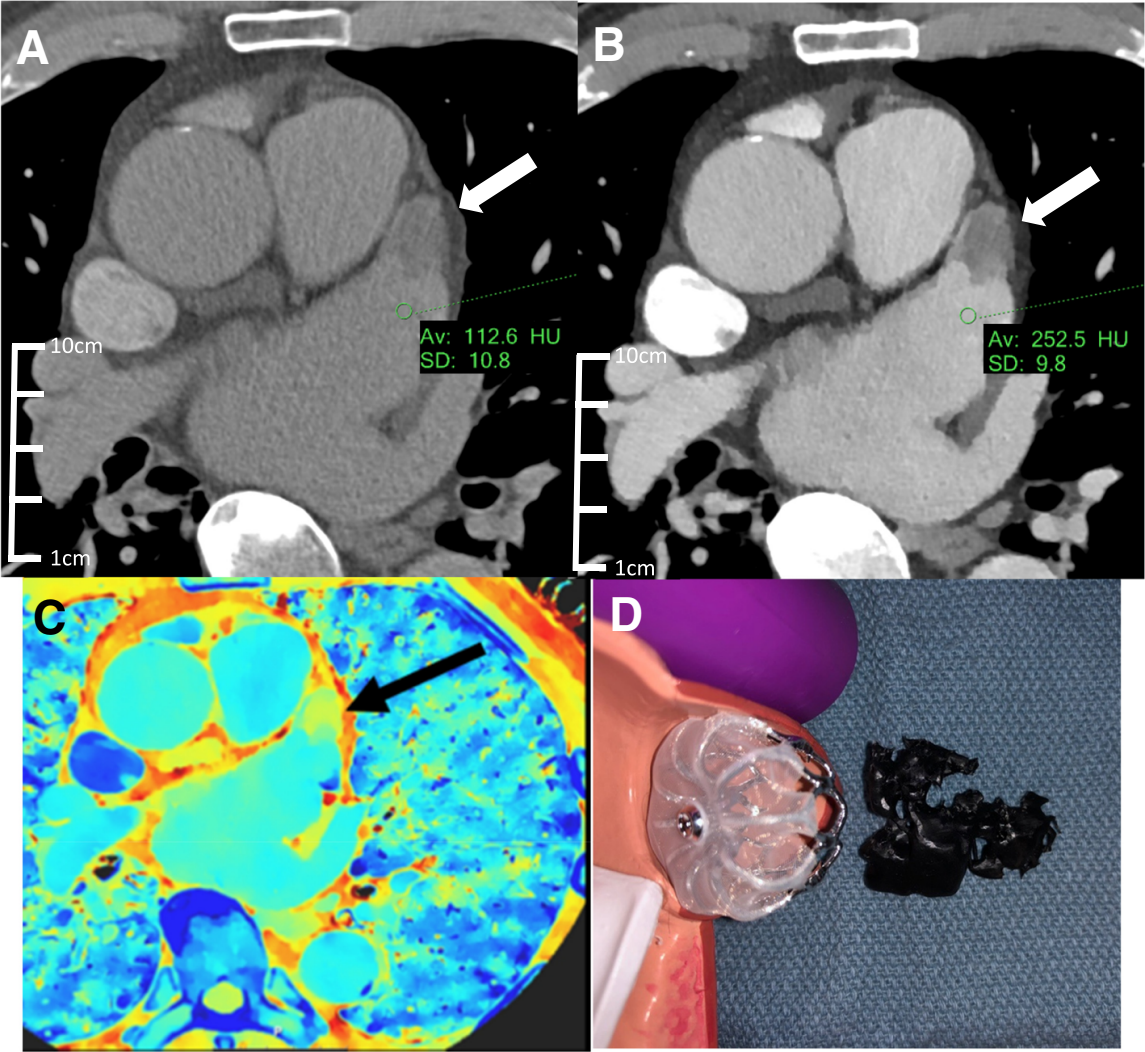
Conventional (**a**) and VMI 40 keV (**b**) axial SDCT delayed contrast phase images of case 2 left atrial appendage thrombus. The left atrial appendage thrombus (arrow) is better demarcated in the 40 keV VMI compared to the conventional CT. Z_effective_ SDCT image (**c**) at the same level shows the effective atomic number value at every voxel which is derived from the photo and scatter values computed from the low and high energy signals. The change in relative atomic number of the thrombus area (arrow, yellow material) relative to the surrounding iodine enhanced material (teal and blue colors) further validates the thrombus composition rather than poor/slow blood flow. (**d**) The 1:1 scale 3D printed LAA thrombus using 40 keV VMI data and the Formlabs Form 2 standard black resin is shown next to an LAA closure device model for scale

Conventional arterial phase images (not shown) demonstrate a filling defect in the anterior aspect of the left atrial appendage (LAA), which may be related to thrombus or circulatory stasis. To confirm and better delineate the thrombus, a 30 s delayed conventional CT scan (Fig. 3a) is obtained, which poorly demonstrates a persistent LAA filling defect, consistent with thrombus.

A 3D printed model was requested for patient education. However, the scan had a very washed out appearance and segmentation of the thrombus using conventional CT data only did not yield an accurate model due to limited attenuation differentiation. The corresponding 40 keV VMI from the delayed phase (Fig. 3b) elegantly demonstrates a thrombus in the left atrial appendage. There was a two-fold increase in HU values between the conventional and VMI target anatomy. Using the 40 keV VMI data set, the thrombus volume was well defined, and the resulting 3D printed model was anatomically 1:1 scale (Fig. 3d).

### Case 3: Malignant tracheal lesion identification and segmentation for resident physician education

**Fig. 4:**
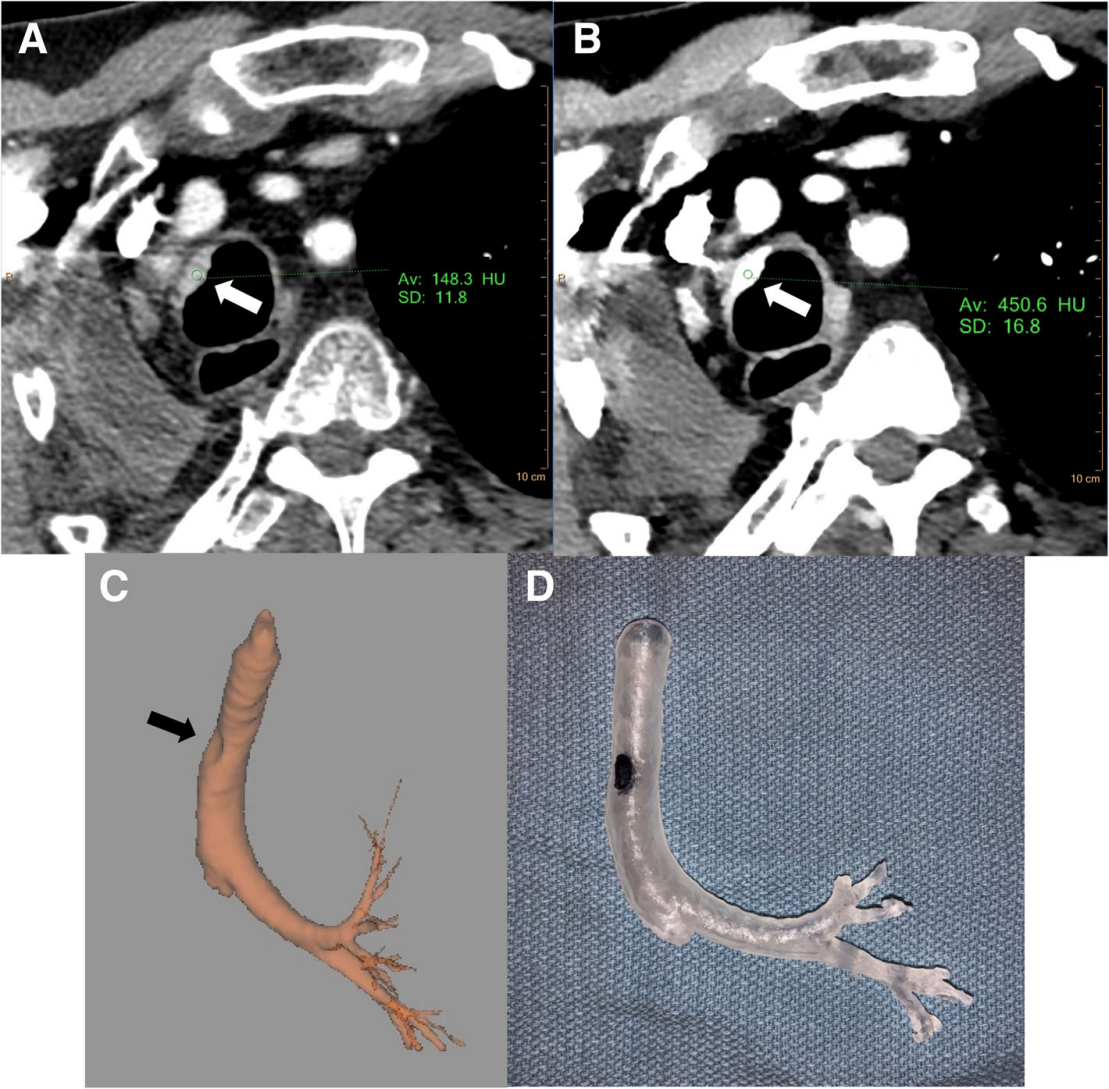
Conventional (**a**) and VMI 40 keV (**b**) axial SDCT images of case 3 malignant tracheal lesion. The recurrent tracheal malignancy (white arrow) is enhanced in the 40 keV VMI compared to conventional CT. 3D volume rendering and segmentation of the bronchial tree from the 40 keV VMI data (**c**) also demonstrates good definition of the tracheal lesion (black arrow). (**d**) 3D printed bronchial tree from the 40 keV VMI data at 50% scale. The Formlabs Form 2 with standard clear resin was used and the tracheal lesion indentation was marked with black ink for visualization

64-year-old male with a history of stage IIA (T1bN1M0) squamous cell carcinoma of the lung status post right pneumonectomy and chemotherapy who was found to have a focal upper right tracheal abnormality on his two year follow up routine CT Chest with contrast (90 mL intravenous Optiray 350) (Fig. 4a). Originally, this was suspected to be adherent mucous within the trachea. However, closer analysis using the VMI data reconstructions revealed enhancement and iodine accumulation, which was highly suspicious for neoplasm recurrence (Fig. 4b). He subsequently underwent bronchoscopy and pathology revealed squamous cell carcinoma suspected to be local regional recurrence versus a new primary malignancy.

A 3D model was requested for operative planning and resident physician education. Segmentation of the airway with the proximal tracheal lesion was technically feasible on both conventional and VMI data. However, there was a subjective decrease in segmentation time when using VMI. Additionally, there was a subjective increase in confidence when selecting the appropriate margins of the tracheal lesion with respect to the surrounding anatomy due to the significant increase in HU differentiation between conventional and VMI.

### Case 4: Salvaged segmentation of left upper extremity trauma with orthopedic hardware artifact

45-year-old-male with history of trauma to the left upper extremity after involvement in a motor vehicle accident. The patient underwent open reduction internal fixation of the left proximal surgical neck and distal humeral shaft fractures with intramedullary rod nailing. After returning ten weeks post-operatively, the patient’s range of motion had improved. However, there was residual left shoulder pain and migration of the proximal hardware screw. A SDCT without contrast of the left upper extremity was performed and demonstrated improved fracture visualization but minimal bony bridging or callus formation.

**Fig. 5:**
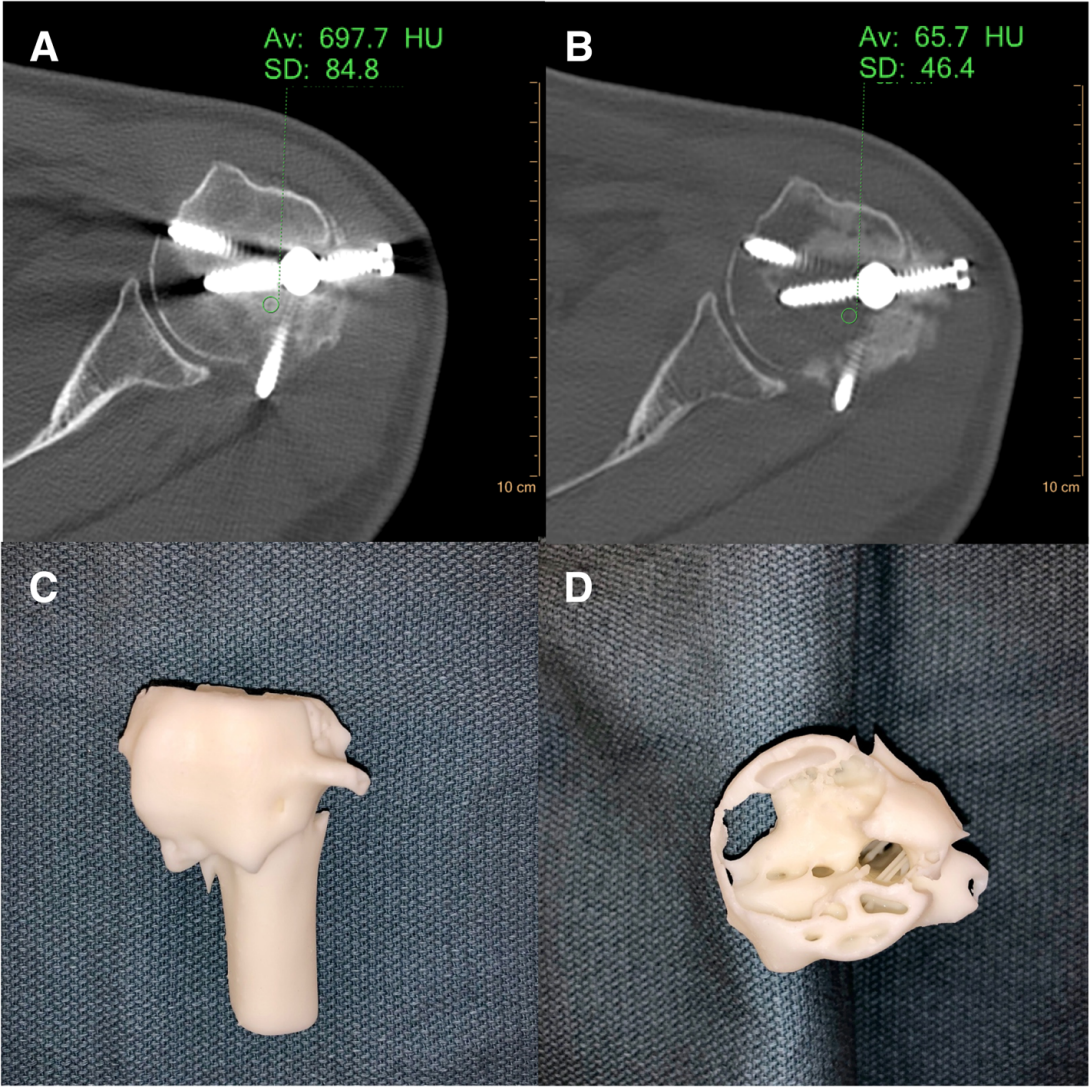
Conventional (**a**) and VMI 120 keV (**b**) axial SDCT images of case 4 left upper extremity trauma. There is significant reduction of metal artifact on the 120 keV VMI allowing for direct visualization of the metal hardware and associated incompletely healed fracture. **c**, **d** Various views of the 3D printed humeral head at 75% scale size using the Formlabs Form 2 standard white resin material. The cross-sectional view through the humeral head (**d**) demonstrates the fracture lines and track from the orthopedic intramedullary hardware

Attempts at segmentation using the conventional CT alone were limited due to the extensive beam hardening artifact originating from the metallic intramedullary orthopedic hardware (Fig. 5a). Using the high 120 keV VMI data, the metal artifact was significantly reduced enabling the auto-segmentation tools to easily differentiate the osseous fracture fragment margins (Fig. 5b). Hounsfield Unit values of the osseous structures adjacent to the metal hardware were reduced by 90% on high keV VMI compared to conventional CT. The resulting 3D printed model clearly demonstrated the fracture margins and outline of intramedullary orthopedic hardware.

For all four cases, the differential in calculated Hounsfield Units between conventional and VMI data at the region of interest (ROI) for segmentation is listed in Table 1. Comparative and differential calculations were also made between the segmented ROI HU and the adjacent tissue material HU. The average primary pre-processing and segmentation time for each of the cases was approximately 60 min. Additional STL mesh post-processing for each model required an additional 30–60 min. 3D printing time averaged between two to five hours each depending on the case, size, and scale of the models.

**Table 1:** Hounsfield Unit (HU) values of segmented anatomy for conventional and VMI data from all four presented cases including ratio differential. Comparative and differential calculations were also made between the segmented ROI HU and the adjacent tissue material HU

Case	Conventional HU at ROI (C)	VMI HU at ROI (V)	HU value of tissue surrounding ROI (S)	HU Ratio Difference (C:V)	Δ HU Conventional HU to HU surrounding ROI (C-S)	Δ HU VMI HU to HU surrounding ROI (V-S)
1	89	206	62	1:2.3	27	144
2	308	666	250	1:2.2	58	416
3	148	451	87	1:3.0	61	364
4	698	66	NA*	1:0.11**	NA	NA

*Case 4 was based on CT artifact reduction rather than surrounding tissue ROI differentiation and therefore HU value of tissue surrounding ROI was not calculated

**The Ratio is less than one, because high keV VMI results in decreased ROI attenuation compared to conventional images

## Discussion

Accurate segmentation of desired anatomy from imaging data for the purposes of 3D printing requires using a combination and variety of software tools [1, 2, 4]. All three of the most common semi-automated segmentation tools, global thresholding, edge detection, and region growing, rely on voxel Hounsfield Unit values to differentiate between different tissues and surrounding anatomical structures [5]. Even manual segmentation tools such as cropping and sculpting count on visual acuity to see the greyscale differences between the target anatomy. SDCT provides both conventional and VMI data with a significant difference in Hounsfield Unit values which aids in the automatic and manual segmentation workflow. While other advanced pixel-based software algorithms that do not completely rely on tissue density may be helpful in certain case scenarios, these tools may not be universally applicable to all cases. To the best of our knowledge, there has been no previously published work associating spectral detector computed tomography and medical 3D printing applications.

In all the cases described above, the segmentation process using VMI data was qualitatively reported as easier, faster, and increased the operator’s confidence in obtaining accurate anatomy. Case 4 also demonstrated how imaging artifacts could be reduced on SDCT enabling segmentation and 3D printing from otherwise unsalvageable imaging sources [13–15]. The single segmentation operator commented that without the VMI data in each of the four detailed cases, the requested segmentation may not have been possible and would have resulted in either time-prohibiting manual segmentation or rescanning the desired patient anatomy.

Since the SDCT VMI data is automatically acquired with every scan without requiring an additional protocol selection or intervention, the VMI data is available to aid in segmentation any time after the image acquisition. This key factor is what enabled our team to retrospectively review any case completed on the SDCT for VMI segmentation. The only workflow change is prospectively requesting any clinical 3D printing cases to have their imaging acquired on the SDCT which we have done in our practice setting.

Due to the complexities of the SDCT and VMI data along with the limited institutional resources, we were able to recruit only one senior person for segmentation of the cases with the knowledgebase and software skills required. One of the primary goals of this project was to establish the concept and workflow for integrating dual-energy CT VMI data into medical 3D printing segmentation. Future directions for this project include training and recruiting additional persons for segmentation to formally quantify and evaluate variability in the segmentation process between multiple persons.

One of the limitations of this workflow is that the VMI data is optimized for use in Philips Intellispace Portal 9.0. The 3D segmentation tools in Philips ISP are robust and the ability to use a slider/toggle to dynamically switch between different keV settings is extremely helpful for both automatic and manual segmentation. However, if the workflow requires utilization of different or more advanced segmentation software, the VMI DICOM data must be post-processed and exported individually at each desired keV value (40 keV DICOM dataset, 60 keV DICOM dataset, etc.). Future software advancements to integrate VMI data natively in other platforms would be ideal and allow for greater flexibility in utilizing spectral data on multiple platforms.

## Conclusions

Including SDCT at the image acquisition phase allows for better utilization of segmentation tools during the medical 3D printing workflow. Complex and previously difficult cases where densities could not be separated are better demarcated with the VMI data. With the rapidly rising adoption of both medical 3D printing and dual-energy computed tomography, combining these technologies may lead to more advanced clinical applications such as new algorithms/tools for segmentation or automatic segmentation of 3D printable regions of interest based on dual-energy material properties. Incorporating SDCT acquisition improves the downstream 3D segmentation process and further enhances the medical 3D printing workflow.

## Additional file

Additional file 1: **Figure S1.** (A) Dual energy axial CT abdomen VMI at various keV energy levels. 40 keV VMI demonstrates marked enhancement of iodine containing structures such as vessels and kidneys. (B) X-ray mass attenuation coefficient (cm^2^/g) of iodine, calcium, and water relative to photon energy (keV). The K-edge of iodine is denoted. **Figure S2.** Variation in segmentation of vascular anatomy using automatic region growing in conventional (A and B) and 40 keV SDCT images (C and D) from the same patient. The blue highlighted area demonstrates the anticipated segmented anatomy relative to the surrounding structures. The automatic segmentation tool overestimates the vascular anatomy in the conventional images (B) due to the poor attenuation differentiation relative to surrounding structures while the same segmentation tool used with 40 keV VMI (D) outlines the vascular lumen properly and even excludes the atherosclerotic calcification. (DOCX 5560 kb)

## Abbreviations

3D: Three-Dimensional
CT: Computed Tomography
HU: Hounsfield Units
IRB: Institutional Review Board
ISP: Philips IntelliSpace Portal
keV: Kilo-Electron Volts
LAA: Left Atrial Appendage
ROI: Region of Interest
SDCT: Spectral Detector Computed Tomography
SLA: Stereolithography
STL: Standard Tessellation Language
TAVR: Transcatheter Aortic Valve Replacement
VMI: Virtual Monoenergetic Images
