# Supplementary material for: Utility of virtual monoenergetic images from spectral detector computed tomography in improving image segmentation for purposes of 3D printing and modeling
Source: 3D Print Med. 2019 Jan 18;5:1. doi: 10.1186/s41205-019-0038-y (PMC6505638; doi:10.1186/s41205-019-0038-y)
Supplement: Supplementary file 1 — Figure S1. (A) Dual energy axial CT abdomen VMI at various keV energy levels. 40 keV VMI demonstrates marked enhancement of iodine containing structures such as vessels and kidneys. (B) X-ray mass attenuation coefficient (cm2/g) of iodine, calcium, and water relative to photon energy (keV). The K-edge of iodine is denoted. Figure S2. Variation in segmentation of vascular anatomy using automatic region growing in conventional (A and B) and 40 keV SDCT images (C and D) from the same patient. The blue highlighted area demonstrates the anticipated segmented anatomy relative to the surrounding structures. The automatic segmentation tool overestimates the vascular anatomy in the conventional images (B) due to the poor attenuation differentiation relative to surrounding structures while the same segmentation tool used with 40 keV VMI (D) outlines the vascular lumen properly and even excludes the atherosclerotic calcification. (DOCX 5560 kb) [file 41205_2019_38_MOESM1_ESM.docx]

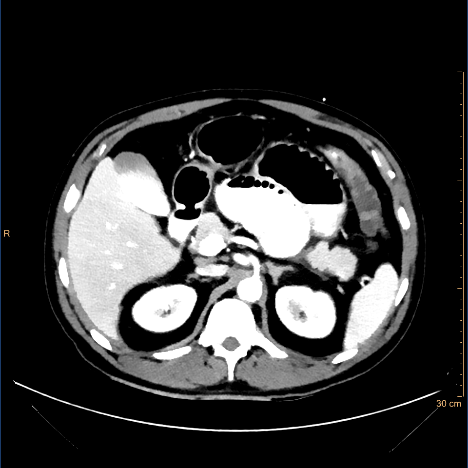

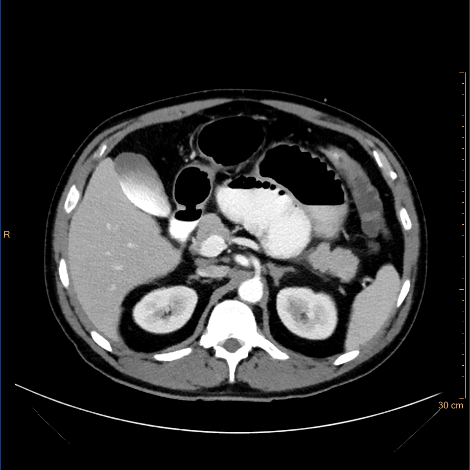

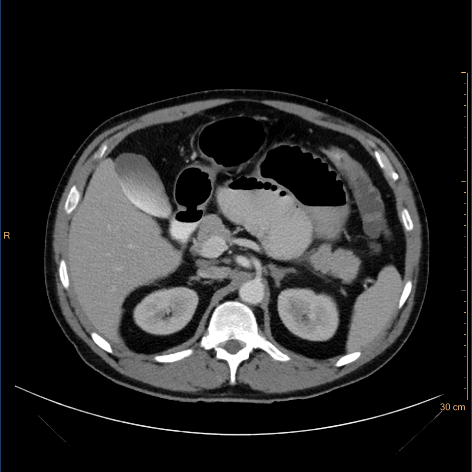

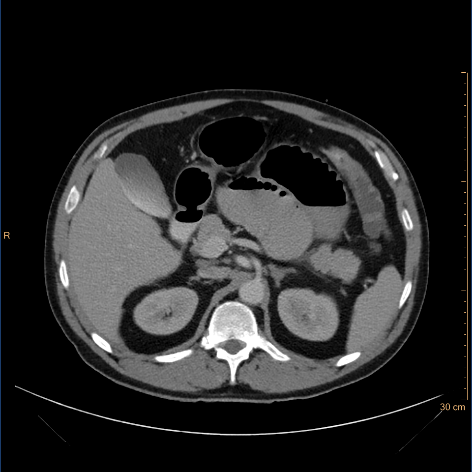

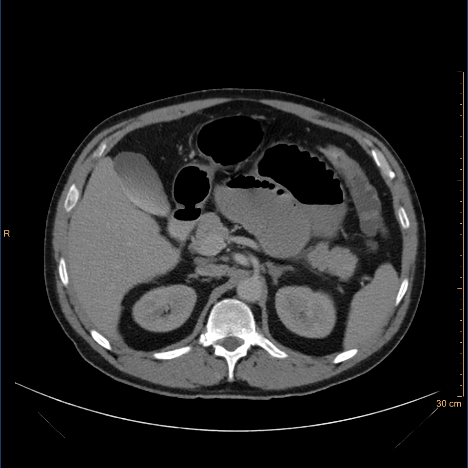

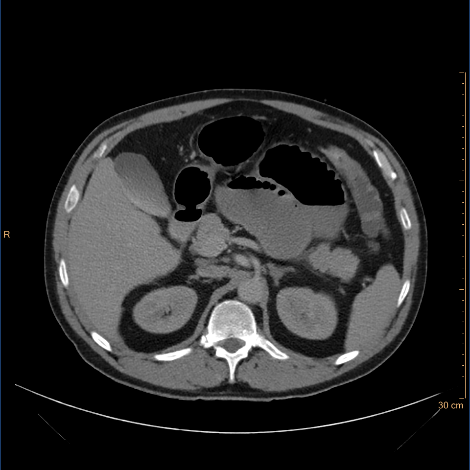

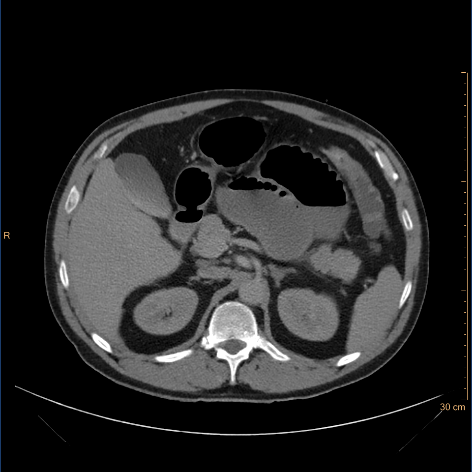

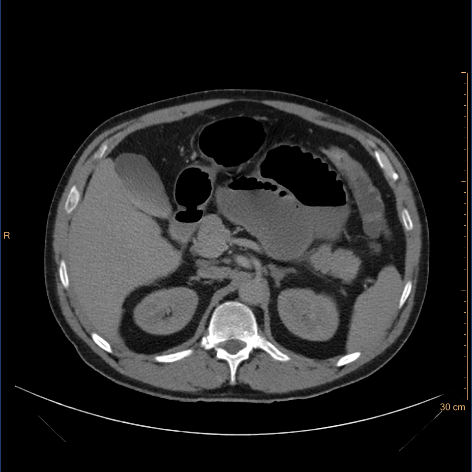


40 keV

60 keV

80 keV

100 keV

120 keV

140 keV

160 keV

180 keV


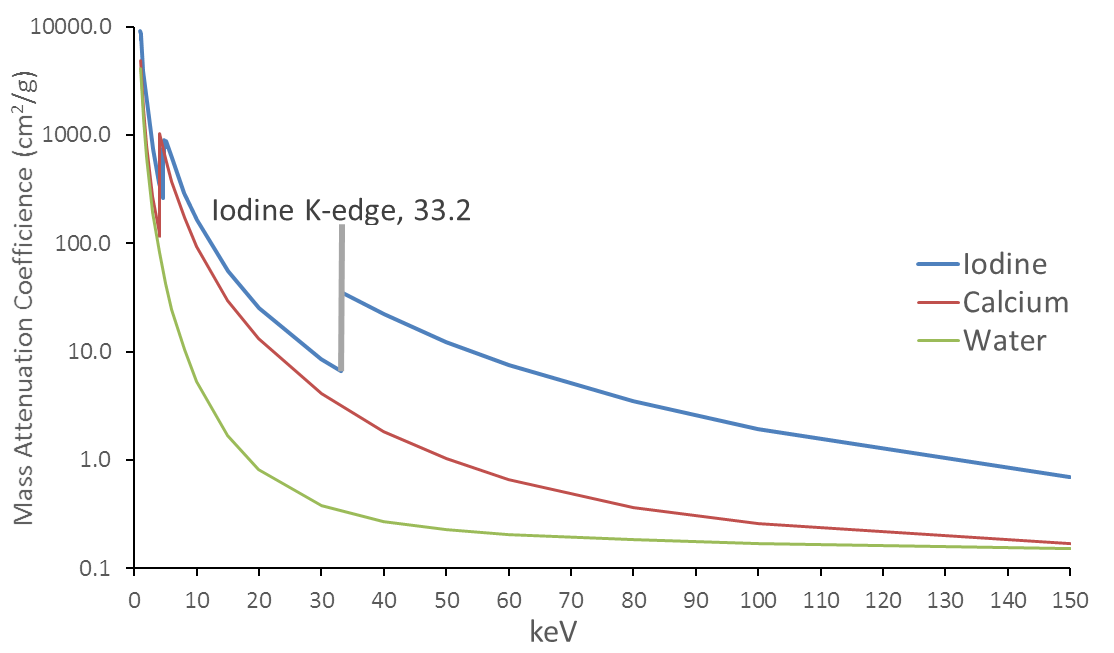


B

A

Supplemental Figure 1: (A) Dual energy axial CT abdomen VMI at various keV energy levels. 40 keV VMI demonstrates marked enhancement of iodine containing structures such as vessels and kidneys. (B) X-ray mass attenuation coefficient (cm^2^/g) of iodine, calcium, and water relative to photon energy (keV). The K-edge of iodine is denoted.

**Supplemental Figure 2:** Variation in segmentation of vascular anatomy using automatic region growing in conventional (A and B) and 40 keV SDCT images (C and D) from the same patient. The blue highlighted area demonstrates the anticipated segmented anatomy relative to the surrounding structures. The automatic segmentation tool overestimates the vascular anatomy in the conventional images (B) due to the poor attenuation differentiation relative to surrounding structures while the same segmentation tool used with 40 keV VMI (D) outlines the vascular lumen properly and even excludes the atherosclerotic calcification.


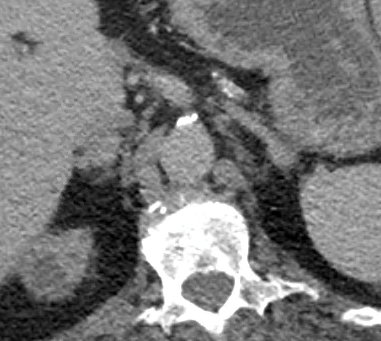

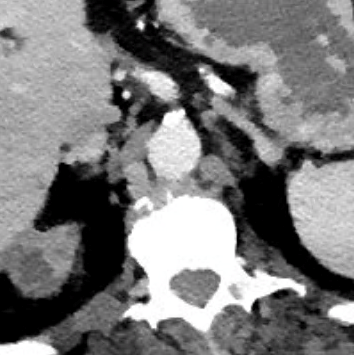

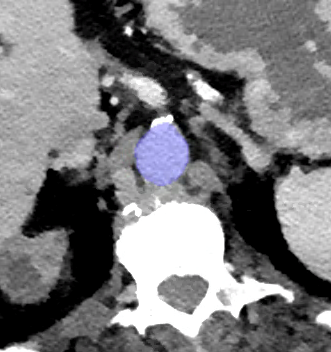

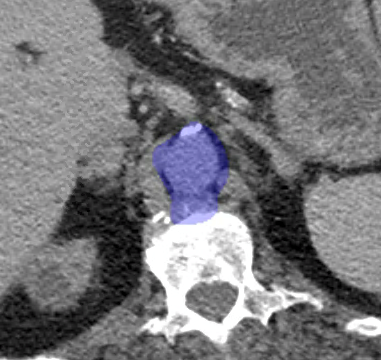


A

B

C

D
